# Genetic Structure of Introduced *Plasmodium vivax* Malaria Isolates in Greece, 2015–2019

**DOI:** 10.3390/tropicalmed9050102

**Published:** 2024-05-02

**Authors:** Ioanna Spiliopoulou, Danai Pervanidou, Nikolaos Tegos, Maria Tseroni, Agoritsa Baka, Annita Vakali, Chrisovaladou-Niki Kefaloudi, Vasilios Papavasilopoulos, Anastasia Mpimpa, Eleni Patsoula

**Affiliations:** 1 European Programme for Public Health Microbiology (EUPHEM), European Centre for Disease Prevention and Control (ECDC), 16973 Stockholm, Sweden; i.spiliopoulou@eody.gov.gr; 2 National Public Health Organization (NPHO), 15123 Athens, Greece; d.pervanidou@eody.gov.gr (D.P.); or mtseroni@nurs.uoa.gr (M.T.); agoritsabaka@gmail.com (A.B.); a.vakali@eody.gov.gr (A.V.); c.kefaloudi@eody.gov.gr (C.-N.K.); 3 National Malaria Reference Center, Laboratory for the Surveillance of Infectious Diseases, Department of Public Health Policy, School of Public Health, University of West Attica, 11521 Athens, Greece; ntegos@uniwa.gr (N.T.); vpapavasilopoulos@uniwa.gr (V.P.); nmpimpa@uniwa.gr (A.M.); 4 Department of Nursing, School of Health Sciences, National and Kapodistrian University of Athens, 123 Papadiamantopoulou Str., Goudi, 11527 Athens, Greece

**Keywords:** *Plasmodium vivax*, genetic structure, microsatellite markers, Greece

## Abstract

Greece has been malaria-free since 1974, after an intense malaria control program. However, as Greece hosts migrant populations from *P. vivax* malaria-endemic countries, there is a risk of introducing the disease to specific vulnerable and receptive areas of the country. Knowledge of the genetic diversity of *P. vivax* populations is essential for understanding the dynamics of malaria disease transmission in a given region. We used nine highly polymorphic markers to genotype 124 *P. vivax*-infected archived DNA samples from human blood specimens referred to the NMRL from all over Greece throughout 2015–2019. The genotypic variability of the samples studied was noted, as they comprised several unique haplotypes, indicative of the importation of a large number of different *P. vivax* strains in the country. However, only a few events of local transmission were recorded. Genotyping revealed and confirmed the same clusters as those identified through epidemiological investigation. In only one introduction event was the index case found. No sustained/ongoing malaria transmissions in/between the studied regions or during consecutive years or additional foci of local transmission were observed. Genotyping is an important component in assisting malaria surveillance, as it provides information concerning the patterns of introduction and the effectiveness of implemented malaria control and elimination measures.

## 1. Introduction

Malaria is one of the world’s major public health problems, causing 249 million infections in 85 malaria-endemic countries and approximately 608,000 deaths in 2022 [1]. It is caused by *Plasmodium* parasites transmitted by the bites of infected females of various *Anopheles* mosquito species. *Plasmodium vivax* (*P. vivax*) is the most geographically widespread human malaria parasite and is the dominant malaria parasite throughout the WHO Region of the Americas and in much of the WHO Eastern Mediterranean Region, Southeast Asia Region, and Western Pacific Region [1]. The global burden of *P. vivax* malaria was estimated at 14 million cases in 2017, and it is concentrated mainly in six countries—Ethiopia, India, Pakistan, Afghanistan, Papua New Guinea, and Indonesia [2]. Although globally the proportion of cases due to *P. vivax* among all malaria cases decreased from approximately 8% (20.5 million) in 2000 to 3% (6.9 million) in 2022, global efforts to control and eliminate malaria [3] have been less successful at reducing the burden of *P. vivax* compared with that due to *P. falciparum*. *P. vivax* has emerged as the dominant species in specific WHO regions after the decline in *Plasmodium falciparum* cases worldwide [4]. *P. vivax* presents distinct challenges for control and elimination due to several unique characteristics: its ability to relapse and form long-lasting, dormant liver stages; its high transmission potential caused by early and continuous production of gametocytes; its high infectivity to mosquitoes; its shorter development cycle in the vector compared with other *Plasmodium* species [5]; and its relatively mild clinical course, which makes it harder to detect [6,7]. Patients who do not receive radical treatment are susceptible to relapses, months, and even years after the first infection, while an additional challenge is the emergence of drug resistance to chloroquine [8] in multiple WHO regions. Finally, the discovery of severe and even fatal cases due to *P. vivax* calls into question the benign status of *P. vivax* malaria [9].

Knowledge of the genetic diversity of *P. vivax* populations is important for understanding the dynamics of malaria transmission and its epidemiology in different regions, which could help in assessing the effectiveness of malaria control and elimination measures [9,10,11,12]. Furthermore, the use of molecular tools that genetically fingerprint *P. vivax* parasites supports epidemiological case classification (imported or locally acquired/introduced). This is of particular significance in cases where medical or travel history is unclear or inadequate to define the importation status and the case classification, contributing to the identification of unsuspected events of local transmission or the exclusion of local transmission [13]. Analysis of allelic variation at multiple distinct loci provides an effective means to determine population structure [10,14,15]. The gene encoding merozoite surface protein-3α (*Pvmsp-3α*), a blood-stage antigen, has been widely used for genotyping, because this gene is highly polymorphic and under natural selection [13,16,17,18]. In addition, microsatellite (MS) DNA markers are short, tandem, one-to-six-nucleotide repeats that are frequently found throughout the genome and are typically selectively neutral. They provide increased resolution compared with *Pvmsp-3α* and have become an important tool for studying the population diversity and structure, evolutionary history, and multiplicity of infection (MOI) of malaria parasite infections [19,20].

Greece has been malaria-free since 1974, after many years (1946–1960) of intense public health efforts [21]. Since then, a small number of imported cases have been reported annually. However, during 2009–2012, sporadic locally acquired (LA) *P. vivax* malaria cases and a few clusters were recorded in Greece, mainly in rural and wetland areas constituting vulnerable and receptive sites, especially since the presence of adequate numbers of Anopheles genus mosquitoes (e.g., *Anopheles sacharovi, A. superpictus*, and *A. maculipenis*) and competent malaria vectors combined with high numbers of migrant populations from malaria-endemic countries [21]. Evrotas, an agricultural area in Lakonia in southern Greece with a large migrant population from the Indian subcontinent, was the most affected area, with clusters of cases in 2011–2012 [22]. Since 2012, a number of intense public health measures have been implemented, including enhanced malaria surveillance, active case detection, and treatment of cases, as well as mass chemoprophylaxis during 2013–2015, in cooperation with various stakeholders at the national, regional, and local levels, which has successfully prevented the establishment of malaria in Greece [22,23]. In 2015, the Ministry of Health published the “National Action Plan for the Management of Malaria”. According to these plans, a series of activities are implemented nationwide for the prevention and management of malaria, with the collaboration of national, regional, and local authorities, which include risk assessment, enhancement of malaria surveillance and laboratory malaria diagnosis capacity, proper case management, communication activities for the public, health professionals and local authorities, mosquito vector surveillance and control activities, and blood safety measures [24].

## 2. Materials and Methods

### 2.1. Blood Sample Collection and DNA Extraction

During 2015–2019, 405 malaria cases were reported to the Hellenic National Public Health Organization (NPHO). All cases were investigated in a timely manner and classified according to their importation status (imported and locally acquired/introduced) from the NPHO’s Vector-borne Diseases Department; a total of 372 cases were imported, and 33 cases were classified as locally acquired/introduced (i.e., the first generation of transmission), 32 (97%) of which were *P. vivax* malaria cases [22]. The National Malaria Reference Laboratory (NMRL, School of Public Health, University of West Attica) receives blood samples from any laboratory in Greece for verification of malaria diagnosis and further identification and genotyping of *Plasmodium* species.

**Figure 1:**
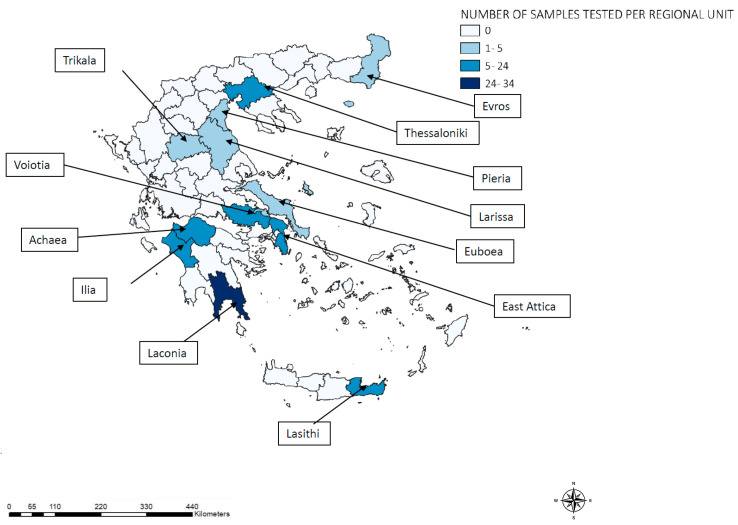
Geographical distribution of 124 *Plasmodium vivax* samples analyzed from imported and introduced cases, Greece, 2015–2019.

We retrospectively studied 124 *P. vivax*-infected archived human blood samples referred to the NMRL from all over Greece, according to the NPHO’s recommendations (Figure 1). Of the 32 *P. vivax* locally acquired/introduced malaria cases in residents of Greece (defined as persons with no travel history to *P. vivax* malaria endemic areas), 30 blood samples were referred to the NMRL. Furthermore, 94 samples were collected from imported cases among migrants from *P. vivax*-endemic countries of the Indian subcontinent, specifically Pakistan (92), Afghanistan (1), and Bangladesh (1). Each sample was derived from a single patient on a single occasion. *P. vivax* infections were confirmed by light microscopic examination of a blood smear, a rapid diagnostic test (RDT), and PCR analysis, as previously described [25]. Parasitic DNA was isolated from 500 μL of peripheral blood using the Maxwell^®^ RSC Whole Blood DNA Kit in the Maxwell RSC automated system (Promega Corporation, Madison, WI, USA).

### 2.2. Genotypic Analysis

Genotypic analysis was performed on blood samples obtained from malaria patients—either hospitalized or during active case detection activities—in areas where *P. vivax* introduction was recorded in the current or previous years [25] or in rural areas with more than one case recorded among migrants from endemic countries, where further investigation is needed to exclude or identify any possible links/clusters among them and validate their importation status.

The *P. vivax* populations were subjected to genotyping targeting 9 main polymorphic markers: the *Plasmodium vivax* merozoite surface protein-3a (*Pvmsp-3a*) locus and 8 microsatellite loci (Pv1.501, Pv3.502, MS1, MS5, MS7, MS8, MS12, and MS20).

Allelic diversity at the *Pvmsp-3a* locus was investigated using a polymerase chain reaction (PCR) protocol as previously described [16]. Gel electrophoresis of the PCR products was carried out in a 1.2% (*w/v*) agarose gel (PanReac Applichem GmbH, Darmstadt, Germany), and the bands were visualized under UV illumination. The amplicons were classified according to size as type A (~1900 bp), B (~1500 bp), or C (~1150 bp).

Subsequently, we performed PCR-based amplification of 8 polymorphic microsatellite markers distributed among 6 of the 14 chromosomes of *P. vivax*. The PCR protocol for the microsatellite loci Pv1.501 and Pv3.502 was performed as previously described [10], while MS1, MS5, MS7, MS8, MS12, and MS20 were amplified according to the protocol described by Karunaweera et al. [15]. All PCR reagents were supplied by Invitrogen (Thermo Fisher Scientific, Waltham, MA, USA), and oligonucleotides were obtained from Eurofins Genomics (Luxembourg). Amplifications were carried out in a GeneAmp PCR system 9700 thermal cycler (Applied Biosystems, San Francisco, CA, USA). The PCR products for all microsatellite markers were analyzed by capillary electrophoresis on an ABI 3730xl DNA analyzer (Applied Biosystems, San Francisco, CA, USA), and Peak Scanner v2 and GeneMapper v4.1 software (Applied Biosystems, San Francisco, CA, USA) were used to measure an allele at each locus compared with the Genescan 500 LIZ size standard (Applied Biosystems, San Francisco, CA, USA).

Because blood-stage malaria parasites are haploid, the presence of >1 allelic variant with a peak height of at least one-third of the main allelic variant peak at a particular locus was interpreted as a coinfection with two or more genetically distinct clones (multiple clone infections) in the same isolate. Haplotypes were defined as unique combinations of alleles at each locus analyzed. Only the predominant alleles were considered for haplotype assignment in multiple-clone infections. Invalid results, defined as <7 of the initial 9 genetic markers multiplied, were excluded from further analysis. We regarded two haplotypes as being the same only when all 9 markers shared the same allelic variant. Samples that shared ≥7 out of the 9 markers examined were suggestive of a distinct family [13,18].

### 2.3. Ethics Statement

This study was conducted in accordance with the Declaration of Helsinki and approved by the Research Ethics Committee of the Hellenic National Public Health Organization (NPHO) (date of approval: 7 June 2023). Patient records were coded and deidentified prior to analysis. No identifying details are included in this article.

## 3. Results

A total of 32 introduced *P. vivax* cases were recorded during the 2015–2019 period in rural areas in Greece; 30 (94%) samples were available for genotyping (one case was diagnosed abroad, and one case was diagnosed only by rapid diagnostic testing). The number of recorded introduced *P. vivax* malaria cases and samples genotyped by the probable Region and Regional Unit of exposure and year of infection are presented in Table 1. All 124 samples referred to the NMRL were successfully genotyped. No invalid results were recorded.

**Figure 2:**
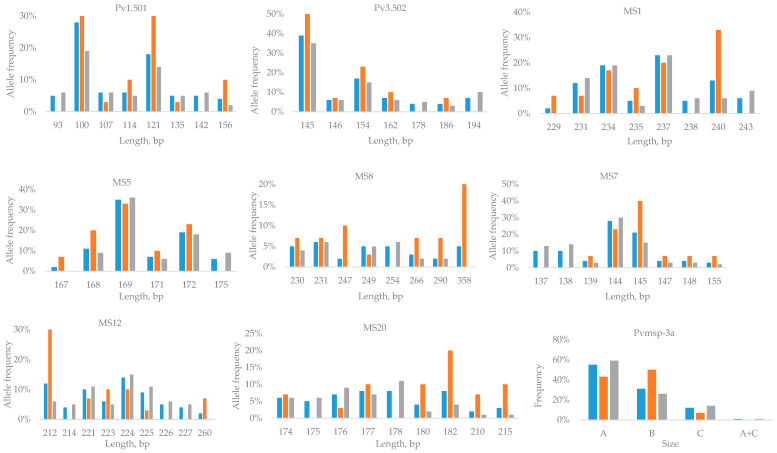
Distribution of the most frequent *P. vivax* allelic variants and lengths or types of genetic markers in Greece’s residents and in migrants, Greece, 2015–2019. The total number of samples is denoted in blue, samples from Greece’s residents are denoted in orange, and samples from migrants are denoted in gray.

The extent of allelic diversity observed differed for the 8 studied polymorphic microsatellite markers (ranging from 16 to 52 distinct variants/markers), with the microsatellite marker Pv3.502 presenting the lowest number of distinct variants and the microsatellite marker MS8 presenting the highest (Table 2). The polymorphic microsatellite markers corresponding to the introduced *P. vivax* malaria cases exhibited less diversity (ranging from 6 to 18 distinct variants/markers) than those from imported cases among migrants (ranging from 16 to 43 distinct variants/markers) (Table 2). The distribution of the most frequent alleles per genetic marker is shown in Figure 2. The allelic frequency distributions differed in Greece’s residents and in migrants, with a few alleles identified either only in Greece’s residents or in migrants. All studied microsatellite loci showed a high frequency of (rare) alleles (frequency lower than 5%), ranging from 59% to 90% (Figure 2). The same trend was observed for both Greece’s residents and migrants, although in migrants, a greater frequency of rare alleles was identified for all markers except MS12. Overall, the *Pvmsp-3a* gene of *P. vivax* was successfully amplified in all but one sample from a migrant (99%). All three allele types (A, B, and C) were detected. Among the introduced *P. vivax* malaria cases, type B was predominant (50%), followed by type A (43%) and type C (7%), while among the imported cases, type A prevailed (59%), followed by type B (26%), type C (14%), and a mixed A and C genotype (1%).

Mixed clone infections were detected in 29 samples (23%), 6 from residents of Greece (20%) and 23 from migrants (24%). In total, 3 out of 29 presented with an allelic variant in two genetic markers, whereas 2/29 samples presented with an allelic variant in three genetic markers. The microsatellite loci presenting the highest multiple clone infections differed among Greece’s residents—MS12 (3/6), MS20 (3/6), and MS7 (2/6)—and among migrants—Pv1.501 (10/23), Pv3.502 (6/23), and MS5 (5/23).

A total of 119 distinct haplotypes were observed. All nine genetic markers were amplified in 93 haplotypes, while 26 haplotypes corresponded to samples in which one (n = 21) or two (n = 5) genetic markers failed to amplify. Genotyping of the 30 recorded introduced *P. vivax* malaria cases revealed 25 distinct haplotypes, with only 2 samples failing to amplify 2 microsatellite markers each.

Of the 30 genotyped *P. vivax* malaria cases introduced, 18 belonged to six families, each comprising between two and seven isolates (Table 3). The remaining introduced *P. vivax* malaria cases were sporadic cases comprising unique haplotypes. The index-imported case was identified for only one introduced *P. vivax* malaria case.

### Genotyping Results per Region

A total of 12 samples from the Attica region were genotyped (including samples from two introduced cases): 7 from 2015, 1 from 2016, and 4 from 2017. Two introduced *P. vivax* malaria cases with an epidemiological link (same family) were recorded in 2015 in the regional unit of East Attica, with haplotypes (Att5 and Att6) and symptom onset on the same date. The haplotypes genotyped from other imported and introduced malaria cases throughout the country during the same year were all distinct from those detected in these two introduced cases. Furthermore, all the haplotypes detected in this Regional Unit in the following years were always distinct. No other introduction event was recorded in the following years in this area.

A total of 27 samples were genotyped from patients from the region of Central Greece (including one sample from an introduced case): 11 samples from 2016 and 16 samples from 2017. The majority of the samples (24/27) were derived from the regional unit of Voiotia, where 2 introduced *P. vivax* malaria cases were recorded—1 in 2015 (diagnosed abroad) and 1 in 2017—with available samples. In 2017, during the focus investigation/reactive case detection following the notification of the introduced *P. vivax* case, the blood sample taken from a migrant residing in the same village had a closely related haplotype (Voi22) belonging to the same family as the haplotype of the introduced *P. vivax* (Voi22-1), indicating that the migrant was likely the index case (Table 3). The haplotypes genotyped from other imported and introduced malaria cases throughout the country were all distinct from these haplotypes. 

A total of 11 samples from patients residing in the region of Central Macedonia were genotyped (including samples from 10 introduced cases), 2 from 2016, 7 from 2018, and 2 from 2019. Two introduced *P. vivax* cases were recorded in 2016 from the regional unit of Thessaloniki, with an epidemiological link (same village) and the same date of symptom onset, for which—although they did not belong to the same family—the relevant haplotypes (Thess1 and Thess2) were highly similar (2/9 genetic markers were the same, and 4/9 differed by only one nucleotide). In 2018, a cluster of eight introduced cases occurred in a Roma settlement, with symptom onset occurring in all patients within three weeks. Samples were available from seven patients (as one patient was diagnosed only by a rapid test). Genotyping revealed three highly similar haplotypes (Thess3 in five patients, Thess3-1, and Thess4) belonging to the same family (Table 3). However, they were distinct from the 2016 haplotypes. In 2019, one introduced *P. vivax* case was recorded in the regional unit of Pieria. Although its haplotype (Pier1) was not the same as the haplotype of a migrant (Pier2) with a *P. vivax* relapse residing in a nearby village, it was highly similar (same 6/9 genetic markers).

In the region of Eastern Macedonia and Thrace, two introduced *P. vivax* cases were recorded in 2018 from the regional unit of Evros in two distinct villages near the Greek–Turkish land borders (with many migrants entering the country) and were genotyped. One patient was infected close to an area where two introduced *P. vivax* cases had been recorded in 2013; genotypic analysis revealed a different haplotype of this case (Evr1) compared with the previously reported introduced cases. The second introduced *P. vivax* case in 2018 also presented a different haplotype (Evr2).

Regarding the region of Thessaly, eight locally acquired *P. vivax* cases were recorded during the study period: four from the regional unit of Larissa, two from the regional unit of Trikala, one from the regional unit of Karditsa, and one from the regional unit of Magnesia and Sporades. All cases’ samples were available and genotyped. The four locally acquired *P. vivax* cases recorded in the regional unit of Larissa during 2015–2016 resided and were considered infected in the same village (where a migrant detention center was located). Three out of these four cases exhibited two similar haplotypes (Lar1 and Lar2) and belonged to the same family; one had symptom onset in 2015, and two had symptom onset in 2016 and were considered infected in 2015 in the same village (where both resided for a short period in 2015 and then left). The fourth case residing in the same village with symptom onset in 2016 had a different haplotype (different introduction). The two recorded introduced malaria cases from the regional unit of Trikala, one in 2015 and one in 2018, resided in the same village (where the migrant population from *P. vivax* malaria-endemic countries also resided) but involved different introductions, as genotypic analysis revealed two different haplotypes (Trik1 and Trik2). Genotyping of one introduced case in the regional unit of Karditsa revealed a haplotype (Kar1) different from the cluster identified in 2012 in another village of the same regional unit [13]. The case recorded from the regional unit of Magnesia and Sporades had a unique haplotype (Spor1).

Six locally acquired *P. vivax* cases were recorded during the study period in the region of western Greece—three from the regional unit of Ilia, two from the regional unit of Achaea, and one from the regional unit of Aitoloakarnania. Eighteen samples were genotyped in total (including samples from the six introduced cases): eight from 2016, nine from 2017, and one from 2018. In the regional unit of Ilia, one introduced *P. vivax* case was notified in 2016, with a different haplotype (Il1) from those identified in two imported cases among migrants (Il2 and Il3) diagnosed in the broader area earlier that year. In 2017, in another nearby town of the regional unit of Ilia, two locally acquired *P. vivax* cases were recorded, with an epidemiological link (family/roommates). Genotyping revealed that the identified haplotypes (Il4 and Il6) of these cases belonged to the same family and were highly similar to the haplotype (Il1) of the notified case in 2016. No other cases were recorded during reactive case detection or in the following years from this area. In the regional unit of Aitoloakarnania, one introduced *P. vivax* case was recorded in 2017 with a unique haplotype (Ait1), with no further cases recorded in the following years. In the regional unit of Achaea, one locally acquired *P. vivax* case was recorded in 2016, and another was recorded in 2017 from the same village. Both haplotypes (Ach1 and Ach6) were highly similar and belonged to the same family but differed from haplotypes from samples collected (passively or actively) from imported cases among migrants from the same regional unit during 2016 and 2017.

Concerning the region of Peloponnese, a total of 35 samples were genotyped: 34 from the regional unit of Laconia, where proactive malaria case detection (PACD) activity has been established, in the Evrotas municipality since 2011 [23] (following the recording of clusters of locally acquired cases), and one from the regional unit of Argolis. Only one introduced *P. vivax* case was recorded in 2015 from the regional unit of Laconia, in a village where locally acquired cases occurred in 2011 and 2012. The haplotype (La6) after genotyping was different from that of previous locally acquired cases [13], indicating that this was a new introduction. Furthermore, there was no genotyping association between this introduced *P. vivax* case and the imported *P. vivax* cases recorded from this area during the study period, as the analyzed haplotypes were unique and distinct. This implies that several distinct introductions of *P. vivax* have occurred in this area.

Νο locally acquired *P. vivax* cases were reported during the study period in the region of Crete. To investigate possible connections with the notified imported *P. vivax* malaria cases among migrants residing in nearby villages in the regional unit of Lasithi, nine samples were genotyped: two from 2015, six from 2016, and one from 2017; all identified haplotypes were distinct (Las1-Las9).

## 4. Discussion

Since 2009, a number of locally acquired/introduced *P. vivax* malaria cases have been detected in some rural areas of Greece (i.e., among patients without travel history to a malaria endemic country), mainly as sporadic introduced cases (first-generation transmission) but also in clusters (mainly in 2011–2012, in Evrotas area). The *Plasmodium* transmission was mosquito-borne through mosquitoes that were infected from imported cases. Following the 2011–2012 peak and the implementation of strict public health measures, the number of locally acquired *P. vivax* cases decreased substantially, despite the increased migrant influx. Since 2013, all locally acquired cases were classified as (probably) introduced (or “cryptic” malaria cases, a term used by others), despite the fact that the index imported cases were not identified; the underdiagnosis of imported *P. vivax* cases among migrants is probably due to the usually mild clinical course of *P. vivax* relapses and challenges regarding the access of undocumented migrants to health care, which mitigates medical care seeking. In this context, in malaria-free Greece, these events of local transmission are considered introduction events, i.e., the first generation of transmission, as intensive reactive case detection and actively enhanced awareness of local clinicians confirmed that no local ongoing transmission occurred in any of these cases during the study period and afterward. The genotyping results also validate the epidemiological conclusion that no ongoing local transmission occurred in the abovementioned cases.

Polymorphic genes are often used to estimate the complexity of parasite populations and reflect transmission intensity. In this study, we successfully determined the genetic structure of locally acquired/introduced *P. vivax* malaria cases recorded in Greece during the period 2015–2019, as sporadic single cases or in small clusters, using nine highly polymorphic markers. Three *PvMSP-3α* allele sizes were identified in this study, similar to the findings of a prior analysis of an earlier collection of samples from Greece in 2009–2013 [13]. The results are also concordant with alleles observed in India [26], Papua New Guinea [27], Thailand [28,29], Afghanistan [30], and Pakistan [17,31,32].

Microsatellite genotyping revealed that the level of genetic variability was highly variable among the studied *P. vivax* population, with 16–52 alleles per locus. Since Greece is a malaria-free country, the number of locally acquired/imported cases among residents was low, and the limited observed allelic diversity was similar to the findings of a previous Greek study [13]. Furthermore, the studied microsatellite markers exhibited a high frequency (59–90%) of rare alleles, much higher than in a study from Venezuela, where the frequency of rare alleles in the same six out of the eight studied microsatellite markers ranged up to 20% [19]. The microsatellite markers in migrants exhibiting high allelic richness were MS8 and MS12, in line with data from Pakistan [9]. Usually, in endemic areas where transmission rates are high, extensively diverse variants are in circulation [17], which is consistent with our observation of greater diversity in imported *P. vivax* strains derived from migrants originating from the Indian subcontinent during the study period.

Multiple clone infections are often observed with *P. vivax* infection, which are caused either by a single mosquito bite carrying a mixture of parasites or by different mosquito bites each taking a single clone [14,33,34]. This provides a surrogate indicator of the level of transmission within populations, as well as the opportunity for recombination between different malaria clones [10]. The percentage of mixed infections (23%) was in line with a previous Greek study [13] and did not differ between Greece’s residents and migrants. Only one isolate (0.80%) exhibited mixed-strain infection regarding the *Pvmsp-3a* locus, a very low percentage in line with other studies from Pakistan, Afghanistan, and Iran [30,31,32,35]. Almost all mixed infections (28/29) involved the microsatellite markers studied. Worldwide, the percentage of mixed infections varies according to the panel of microsatellites used and the geographical region in which the samples originated [11]. In studies using the same six microsatellite markers, as in our study, the available *P. vivax* samples displayed mixed infections, ranging from 15.9% [19] to 45.2% [36].

In the present study, the combined use of the *Pvmsp-3a* locus and 8 microsatellite loci for genotyping 124 *P. vivax* malaria samples identified 119 distinct haplotypes, implying that several distinct introductions of *P. vivax* have occurred in various areas of the country; however, transmission in the studied areas is limited. Specifically, genotyping of the 30 introduced *P. vivax* malaria cases revealed 25 distinct haplotypes, with 18 samples belonging to 6 families, indicating local transmission (first generation of transmission) of prevalent alleles. A few small family or village clusters of introduced cases were recorded during the study period. The clusters revealed through genotyping were in line with and confirmed the clusters originally identified through the epidemiological investigation performed following the recording of each locally acquired *P. vivax* case. One of the aims of the epidemiological outbreak investigations was to identify the index case, which was feasible in only one case (cluster “Lef”), where the haplotypes from both the introduced *P. vivax* malaria case and a migrant residing in the same area belonged to the same family (family 5). As mentioned before, the underdiagnosis of mild imported *P. vivax* cases and relapses is not unexpected.

No sustained/ongoing malaria transmission in/between the studied regions or during consecutive years was observed based on the circulation of different haplotypes throughout the study period, and no additional “foci” of local transmission were identified during the study period.

Genotyping validated the epidemiological hypothesis regarding the likely place of exposure of some cases, revealed a small cluster of three cases (infected in 2015 in the Larissa regional unit, Thessaly), and excluded the association between two cases from the same village in different years (in the Trikala regional unit, Thessaly), confirming the distinct introductions.

Specifically, in the regional unit of Larissa, three cases were recorded (one in 2015 and two in 2016), which exhibited two similar haplotypes (Lar1 and Lar2) and belonged to the same family. The two locally acquired *P. vivax* cases with symptom onset in 2016, residing in different villages in 2016, were considered to have been infected during 2015 in the same village as the 2015 case, as they both lived there in 2015 and were thought to have experienced a long incubation period. Prolonged incubation or latency periods between the initial illness and relapse, lasting 8–12 months, have been reported in the literature for *P. vivax* infections [6,37,38,39]. The hypothesis of a long incubation period was also considered for one introduced *P. vivax* case in the regional unit of Trikala, with symptom onset in late April 2019, for whom exposure was considered to have taken place in 2018 (a long incubation period), as the date of symptom onset was too early in 2019 and the climate conditions were not favorable for mosquito breeding. Regarding the cases in the same village in the regional unit of Larissa, the index case was not detected despite the intensive investigation; however, the village hosted a migrant detention center, and the hypothesis was that the index case was probably a migrant from this center. A fourth introduced case was recorded in late 2016 from the same village with a distinct haplotype, indicating that it involved a distinct introduction.

The two introduced malaria cases from the regional unit of Trikala recorded in 2015 and 2018 originated in the same town. Genotypic analysis of the samples revealed two different haplotypes (Trik1 and Trik2) and confirmed that these cases were not linked.

One limitation of our study is the limited information available regarding the distribution of *P. vivax* alleles circulating in the countries of origin of the imported cases, which challenges the accurate interpretation of the genotyping results. However, based on the genotypic variability of the samples studied during 2015–2019, a relatively large number of different *P. vivax* strains were imported into Greece in various areas of the country.

From a public health perspective, the presence of malaria patients coming from endemic countries, coupled with the existence of competent Anopheles vector populations in rural areas, increases the risk of possible malaria introduction in receptive malaria-free areas of Greece. To support the country’s malaria prevention plan, the genotyping of locally acquired *P. vivax* malaria populations is vital for assisting in malaria surveillance by providing valuable information and contributing to a better understanding of the patterns of introduction and assessing the effectiveness of implemented malaria prevention measures.

## 5. Conclusions

In conclusion, we demonstrated that the introduced *P. vivax* malaria cases recorded during 2015–2019 in Greece largely comprised unique haplotypes, indicating limited transmission in the studied areas. The genotypic analysis confirmed the clusters identified during the epidemiological investigation performed during the study period, with no additional foci of local transmission identified. Detailed knowledge of the genetic diversity of *P. vivax* is useful for implementing and adapting appropriate malaria management measures in affected regions.

## Figures and Tables

**Table 1:** Notified locally acquired/introduced *P. vivax* malaria cases (n = 32) and blood samples from imported (n = 94) and locally acquired/introduced (n = 30) cases genotyped by probable Region and Regional Unit of exposure and year of infection, Greece, 2015–2019.

Region	Regional Unit	Locally Acquired/Introduced (LA/I) *P. Vivax* Cases by Year of Infection and Blood Samples Genotyped from Imported (IMP) and locally Acquired/Introduced (LA/I) Cases														
		2015			2016			2017			2018			2019		
		Cases	Samples		Cases	Samples		Cases	Samples		Cases	Samples		Cases	Samples	
		LA/I	LA/I	IMP	LA/I	LA/I	IMP	LA/I	LA/I	IMP	LA/I	LA/I	IMP	LA/I	LA/I	IMP
Peloponnese	Laconia	1	1	7	0	0	15	0	0	11	0	0	0	0	0	0
	Argolis	0	0	0	0	0	1	0	0	0	0	0	0	0	0	0
Attica	East Attica	2	2	5	0	0	1	0	0	4	0	0	0	0	0	0
Central Greece	Voiotia	1	0 ^a^	0	0	0	8	1	1	15	0	0	0	0	0	0
	Euboea	0	0	0	0	0	2	0	0	0	0	0	0	0	0	0
	Phtiotis	0	0	0	0	0	1	0	0	0	0	0	0	0	0	0
Thessaly	Karditsa	0	0	0	0	0	0	1	1	0	0	0	0	0	0	0
	Larissa	3	3	0	1	1	1	0	0	0	0	0	0	0	0	0
	Trikala	1	1	0	0	0	0	0	0	0	1	1	0	0	0	0
	Sporades	0	0	0	1	1	0	0	0	0	0	0	0	0	0	0
Eastern Macedonia and Thrace	Evros	0	0	0	0	0	0	0	0	0	2	2	0	0	0	0
Central Macedonia	Pieria	0	0	0	0	0	0	0	0	0	0	0	0	1	1	1
	Thessaloniki	0	0	0	2	2	0	0	0	0	8	7 ^a^	0	0	0	0
Western Greece	Achaea	0	0	0	1	1	4	1	1	2	0	0	1	0	0	0
	Ilia	0	0	0	1	1	2	2	2	3	0	0	0	0	0	0
	Aitoloakarnania	0	0	0	0	0	0	1	1	0	0	0	0	0	0	0
Ionian islands	Kefalonia	0	0	0	0	0	1	0	0	0	0	0	0	0	0	0
Crete	Lasithi	0	0	2	0	0	6	0	0	1	0	0	0	0	0	0
**Total**		**8**	**7**	**14**	**6**	**6**	**42**	**6**	**6**	**36**	**11**	**10**	**1**	**1**	**1**	**1**

^a^ DNA samples were not available for genotyping.

**Table 2:** The 8 *P. vivax* microsatellite loci, chromosome number in the Salvador-I/strain, size range of the amplified alleles in base pairs, and number of alleles per polymorphic microsatellite of *P. vivax* malaria cases in Greece’s residents and migrants, Greece, 2015–2019.

Locus	Chromosome Location	Allele Size, bp			Number of Alleles/Locus		
		Total	Greece’s Residents	Migrants	Total	Greece’s Residents	Migrants
Pv1.501	1	82–207	100–185	82–207	31	10	29
Pv3.502	3	89–210	145–210	89–210	16	6	16
MS1	3	155–283	229–246	155–283	17	7	16
MS5	6	112–187	167–184	112–187	19	7	17
MS7	12	137–160	139–160	137–159	17	8	16
MS8	12	216–358	225–358	216–334	52	18	43
MS12	5	169–262	209–262	169–237	27	15	23
MS20	10	168–227	171–215	168–227	39	15	37

**Table 3:** Characteristics of the introduced *P. vivax* malaria cases belonging to the six identified families, Greece, 2015–2019.

Date of Symptoms Onset	Year of Exposure	Region	Regional Unit	Cluster	Introduced ^a^	Pv1.501	Pv3.502	MS1	MS5	MS7	MS8	MS12	MS20	*Pvmsp-3a*	Haplotypes	Family
30 September 2015	2015	Attica	East Attica	village 1	Y	114	186	246	167	147	290	224	195	A	Att5	1
30 September 2015	2015	Attica	East Attica	village 1	Y	114	186	246	167	145	290	215	195	A	Att6	
8 September2015	2015	Thessaly	Larissa	village 2	Y	121	162	229	172	145	247	260	180	B	Lar1	2
11 September 2016	2015	Thessaly	Larissa	village 2	Y	121	162	229	172	145	247	260	180	B	Lar1	
27 October 2016	2015	Thessaly	Larissa	village 2	Y	121	162	231	172	145	247	262	180	B	Lar2	
3 July 2016	2016	Western Greece	Achaea	village 3	Y	100	145	237	172	155	248	221	171	A	Ach1	3
2 May 2017	2017	Western Greece	Achaea	village 3	Y	100	145	237	172	155	250	221	172	A	Ach6	
18 July 2017	2017	Western Greece	Ilia	village 4	Y	121	154	234	168	144	266	223	209	A	Il4	4
4 May 2017	2017	Western Greece	Ilia	village 4	Y	121	154	234	168	144	266	224	210	A	Il6	
15 July 2017	2017	Central Greece	Voiotia	village 5	Y	156	210	237	168	x ^b^	x ^b^	212	177	B	Voi22-1	5
14 July 2017	2017	Central Greece	Voiotia	village 5	N	156	210	237	168	148	249	212	177	B	Voi22	
12–15 September 2018	2018	Central Macedonia	Thessaloniki	village 6	Y	100	145	240	169	145	358	212	182	B	Thess3	6
17 September 2018	2018	Central Macedonia	Thessaloniki	village 6	Y	100	145	240	169	145	357	212	182	B	Thess3-1	
26 September 2018	2018	Central Macedonia	Thessaloniki	village 6	Y	100	145	240	169	145	358	212	182	B	Thess3	
27–28 September 2018	2018	Central Macedonia	Thessaloniki	village 6	Y	100	145	240	169	145	358	212	182	B	Thess3	
2 October 2018	2018	Central Macedonia	Thessaloniki	village 6	Y	100	145	240	169	145	358	212	182	B	Thess3	
3–5 October 2018	2018	Central Macedonia	Thessaloniki	village 6	Y	100	145	240	169	145	358	212	182	B	Thess3	
5 October 2018	2018	Central Macedonia	Thessaloniki	village 6	Y	100	145	240	169	145	358	x ^b^	x ^b^	B	Thess4	

^a^ Introduced: Y = Yes, N = No, ^b^ x = failed to amplify.

## Data Availability

The data presented in this study are available on request.
